# Commensal Bacteria Augment *Staphylococcus aureus* septic Arthritis in a Dose-Dependent Manner

**DOI:** 10.3389/fcimb.2022.942457

**Published:** 2022-07-22

**Authors:** Ying Fei, Abukar Ali, Majd Mohammad, Tao Jin

**Affiliations:** ^1^ Department of Rheumatology and Inflammation Research, Institute of Medicine, Sahlgrenska Academy at University of Gothenburg, Gothenburg, Sweden; ^2^ Department of Microbiology and Immunology, The Affiliated Hospital of GuiZhou Medical University, Guiyang, China; ^3^ Department of Rheumatology, Sahlgrenska University Hospital, Gothenburg, Sweden

**Keywords:** S. aureus, S. epidermidis, S. mitis, septic arthritis, mice

## Abstract

**Background:**

Septic arthritis is considered one of the most dangerous joints diseases and is mainly caused by the Gram-positive bacterium *Staphylococcus aureus* (*S. aureus*). Human skin commensals are known to augment *S. aureus* infections. The aim of this study was to investigate if human commensals could augment *S. aureus*-induced septic arthritis.

**Method:**

NMRI mice were inoculated with *S. aureus* alone or with a mixture of *S. aureus* together with either of the human commensal *Staphylococcus epidermidis* (*S. epidermidis*) or *Streptococcus mitis* (*S. mitis*). The clinical, radiological and histopathological changes due to septic arthritis were observed. Furthermore, the serum levels of chemokines and cytokines were assessed.

**Results:**

Mice inoculated with a mixture of *S. aureus* and *S. epidermidis* or *S. mitis* developed more severe and frequent clinical arthritis compared to mice inoculated with *S. aureus* alone. This finding was verified pathologically and radiologically. Furthermore, the ability of mice to clear invading bacteria in the joints but not in kidneys was hampered by the bacterial mixture compared to *S. aureus* alone. Serum levels of monocyte chemoattractant protein 1 were elevated at the early phase of disease in the mice infected with bacterial mixture compared with ones infected with *S. aureus* alone. Finally, the augmentation effect in septic arthritis development by *S. epidermidis* was bacterial dose-dependent.

**Conclusion:**

The commensal bacteria dose-dependently augment *S. aureus*-induced septic arthritis in a mouse model of septic arthritis.

## Introduction

Although major strides have been made during the last decades in treatment of bacterial infections, septic arthritis still remains a major concern for the wider health-community. Its prevalence is relatively unchanged; prognosis still remains poor with majority of patients suffering from permanent joint-dysfunction even after adequate treatment (Goldenberg, 1998; Tarkowski, 2006).

Most at risk of septic arthritis are immuno-compromised patients, the elderly as well as patients with underlying joint damages, e.g. rheumatoid arthritis (Goldenberg, 1998; Tarkowski, 2006). In fact, the prevalence among patients with underlying joint disease is up to 10 times higher than in the general population (Tarkowski, 2006). The most common causative agent isolated from septic arthritis patients is Gram-positive *Staphylococcus aureus* (*S. aureus*), accounting for almost half of all cases (Goldenberg, 1998; Tarkowski, 2006). However, other Gram-positive- as well as Gram-negative bacteria have been shown to cause septic arthritis, although to a lesser extent (Goldenberg, 1998; Tarkowski, 2006). In line with the emergence and rapid spread of methicillin-resistant *S. aureus* (MRSA), the prevalence of MRSA caused septic arthritis was found to be high (25-50%) in both UK and USA (Al-Nammari et al., 2007; Frazee et al., 2009). Importantly, patients with MRSA septic arthritis tended to be older and have more polyarticular involvement as well as higher mortality compared to patients with non-MRSA septic arthritis (Ross and Davidson, 2005; Al-Nammari et al., 2007).

The development of new treatments for septic arthritis has stagnated. To limit the immune response and reduce the risk of permanent joint destruction, a combination treatment of antibiotics and immunomodulatory therapy was proposed (Fei et al., 2011). However, recent data suggest that there are potential dangers associated with such combination therapies as long as the problem of antibiotic resistance persists (Ali et al., 2015a; Ali et al., 2015b). Understanding the bacterial components/host factors interaction responsible for joint inflammation and destruction is the key for the development of new therapies. It is known that antibiotic-killed *S. aureus* induce destructive arthritis through TNF receptor 1 and that bacterial cell walls are the culprits (Ali et al., 2015c). We have recently shown that *S. aureus* lipoproteins are strongly arthritogenic and cause severe bone destruction by activating monocytes/macrophages in an experimental setting (Mohammad et al., 2019). Interestingly, a recent study demonstrated that the interaction between *S. aureus* coagulases, especially von-Willebrand binding protein and host von-Willebrand factor is crucial for initiation and development of septic arthritis (Na et al., 2020), suggesting the new therapeutic strategies by blocking such bacteria/host interaction.

Recently, we showed that human skin commensals augment the pathogenicity of *S. aureus* (Boldock et al., 2018)*. Streptococcus mitis* (*S. mitis*), a Gram-positive coccus, is part of the oral flora. Although *S. mitis* is usually not associated with septic arthritis, there have been several reported cases of septic arthritis caused by *S. mitis*, most likely due to transmission from health practitioners preforming joint injections (Coatsworth et al., 2013; Feder and Gruson, 2016; Cain et al., 2018). *Staphylococcus epidermidis* (*S. epidermidis*), is another Gram-positive bacterium that is part of the normal human skin flora. *S. epidermidis* is typically considered to be non-pathogenic, but can take advantage of the host’s compromised immune system, causing various infections (Kim and Joo, 2012). Immunosuppressed patients are well-known to be susceptible to the different infections including septic arthritis. Anti-TNF therapy used in rheumatoid arthritis is associated with increased risk of septic arthritis. Interestingly, several opportunistic species that seldom cause septic arthritis were reported in the patients treated with anti-TNF therapy (Galloway et al., 2011). Similarly, opportunistic bacteria such as atypic mycobacterium were found in the joint infections in patients with human immunodeficiency virus (Zalavras et al., 2006), suggesting that immunosuppression may increase the probability of opportunistic bacterial septic arthritis.

Since human commensals have been shown to augment the pathogenicity of *S. aureus* infections such as sepsis, our hypothesis for the current study was to investigate if this would be also true for specific infections, such as septic arthritis. To this end, mice were inoculated with either *S. aureus*, *S. epidermidis*, *S. mitis* or a combination of *S. aureus* and *S. epidermidis* or *S. aureus* and *S. mitis*. Our data demonstrate that both *S. epidermidis* and *S. mitis* augment *S. aureus*-induced septic arthritis.

## Material and Methods

### Mice

Female NMRI mice, 6– 8 weeks old, were purchased from Envigo (Venra, Netherlands). All mice were housed in the animal facility located at the Department of Rheumatology and Inflammation Research, University of Gothenburg. The mice were kept under standard conditions of temperature and light, and were fed laboratory chow and water ad libitum. The ethical committee of animal research of Gothenburg approved the experiments.

### Preparation of Bacterial Solutions

The following bacterial species were used in this study: *S. aureus* LS-1 strain,

*S. epidermidis* strain CCUG 61325, and *S. mitis* strain CCUG 63687. The bacterial suspensions were thawed, washed in phosphate-buffered saline (PBS), and adjusted to the concentration required before conducting the experiments, as previously described (Jin et al., 2019).

### Experimental Protocols for Inducing Septic Arthritis

Several separate *in vivo* experiments were performed to investigate if human commensals aggravate *S. aureus*-induced septic arthritis. In all experiments, NMRI mice were inoculated intravenously into the tail vein with 200 μl of the respective bacterial strain suspensions in PBS. To prepare bacterial mixture, each of the bacterial strains was prepared in PBS in twice as high bacterial concentration and then mixed in a 1:1 ratio volume wise. To study whether *S. epidermidis* aggravated *S. aureus*-induced septic arthritis, mice were inoculated with the following doses: *S. aureus* LS-1 strain, 1.2x10^6^ colony forming units (CFU)/mouse; *S. epidermidis*, CCUG 61325 strain, 1.2x10^8^ CFU/mouse; or Mixture of *S. aureus* LS-1 strain, 1.2x10^6^ CFU/mouse with either *S. epidermidis* 1.2x10^6^, 1.2x10^7^ or 1.2x10^8^ CFU/mouse.

To study whether *S. mitis* aggravated *S. aureus*-induced septic arthritis, mice were inoculated with the following doses: *S. aureus* 2.8x10^6^ CFU/mouse; *S. mitis*, CCUG 63687, 2.4x10^8^ CFU/mouse; or Mixture of *S. aureus* 2.8x10^6^ CFU/mouse with *S. mitis* 2.4x10^8^ CFU/mouse.

The mice were monitored individually and the survival, weight development and clinical arthritis were assessed regularly, as previously described (Mohammad et al., 2020). At the end of experiments, the mice were anaesthetized with ketamine hydrochloride (Pfizer AB, Sweden) and medetomidine (Orion Pharma, Finland) before blood from the axillary artery was collected. Afterwards the mice were immediately sacrificed by cervical dislocation.

### Clinical Evaluation of Arthritis

Observers blinded to the treatment groups visually inspected all 4 limbs of each mouse. Arthritis was defined as erythema and/or swelling of the joints. To evaluate the severity of arthritis, a clinical scoring system ranging from 0-3 was used, as previously described (Bremell et al., 1991; Ali et al., 2015a).

### Bacteriologic Examination

To study whether the bacteria invaded and persisted in the kidneys or joints, mice were inoculated with either *S. aureus* alone, *S. epidermidis* alone or a mixture of both bacterial species in 1:100 ratio, respectively. After sacrificing the mice, the kidneys of the mice were aseptically removed, homogenized, plated on blood agar plates, and quantified as CFUs on day 7 and 10 post-infection, as described elsewhere (Mohammad et al., 2016), while different joint groups (forepaws and wrists, elbows, shoulders, back paws and ankles, knees, and hips) from each animal were collected separately and homogenized for CFU counts on day 7 when the clinical arthritis became evident, as previously described (Na et al., 2020). For mixed infections, the bacteria were identified by morphological characteristics.

### Microcomputed Tomography (Micro-CT)

The mice were sacrificed and joints removed, fixed in 4% paraformaldehyde for 3 days and transferred to PBS for 24 hrs. Thereafter, all limb joints were scanned with Skyscan1176 micro-CT (Bruker, Antwerp, Belgium) with a voxel size of 35 μm. The scanning was done at 55kV/455 mA, with a 0.2 mm aluminium filter. Exposure time was 47 ms. The X-ray projections were obtained at 0.7° intervals with a scanning angular rotation of 180°. The projection images were reconstructed into three-dimensional images using NRECON software (version 1.6.9.8; Bruker) and analyzed with CT-Analyzer (version 2.7.0; Bruker). After reconstruction, experienced observers (Y.F, T.J) evaluated, in a blinded manner, the extent of bone and cartilage destruction on a grading scale from 0-3, as previously described (Ali et al., 2015b; Mohammad et al., 2019).

### Histopathological Examination of Joints

After the scanning, joints were decalcified, embedded in paraffin and sectioned with microtome. Tissue sections were thereafter stained with hematoxylin and eosin. All slides were coded and assessed in a blinded manner by observers (Y.F, T.J). The extent of synovitis as well as cartilage and bone destruction were judged as previously described (Ali et al., 2015c; Mohammad et al., 2019).

### Measurement of Cytokine and Chemokine Levels

The levels of monocyte chemoattractant protein (MCP-1), tumor necrosis factor alpha (TNF-α) and interleukin 6 (IL-6) in mouse blood serum were quantified, using DuoSet ELISA Kits (R&D Systems, Abingdon, UK).

### Statistical analysis

Statistical significance was assessed using the Mann-Whitney U test and Fisher’s exact test as appropriate. Results are reported as the mean ± standard error of the mean (SEM) unless indicated otherwise. A *p* value <0.05 was considered statistically significant. Calculations were performed using GraphPad Prism version 9.3.0 software for Mac (GraphPad software, La Jolla, CA, USA).

## Results

### *S. epidermidis* and *S. mitis* augments *S. aureus*-Induced Septic Arthritis

**Figure 1:**
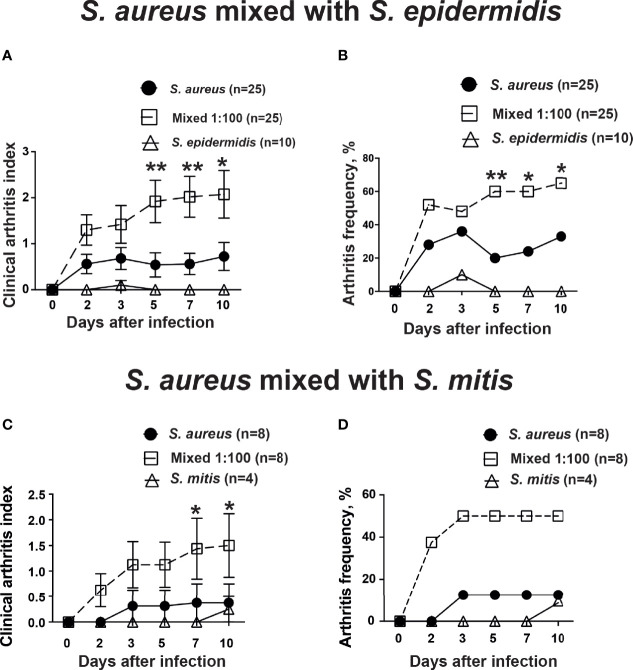
*S. epidermidis* and *S. mitis augment S. aureus*-induced septic arthritis. NMRI mice intravenously inoculated with either *Staphylococcus aureus (S. aureus)* LS-1 strain (1.2 x 10^6^ CFU/mouse) alone, *Staphylococcus epidermidis (S. epidermidis)* CCUG 61325 (1.2 x 10^6^ CFU/mouse) alone or with *S. aureus* LS-1 strain (1.2 x 10^6^ CFU/mouse) mixed with *S. epidermidis* CCUG 61325 (1.2 x 10^8^ CFU/mouse, denoted Mixed 1:100). The clinical severity of arthritis **(A)** and the frequency of arthritis **(B)** were followed for 10 days after infection. NMRI mice intravenously inoculated with either *S. aureus* LS-1 strain (2.8 x 10^6^ CFU/mouse) alone, *Streptococcus mitis (S. mitis)* CCUG 63687 (2.4 x 10^6^ CFU/mouse) alone or with *S. aureus* LS-1 strain (2.8 × 10^6^ CFU/mouse) mixed with *S. mitis* CCUG 63687 (2.4 x 10^8^ CFU/mouse, denoted Mixed 1:100). The clinical severity of arthritis **(C)** and the frequency of clinical arthritis **(D)** were followed for 10 days after infection. Statistical evaluations were performed using the Mann–Whitney U test, with data expressed as the mean ± standard error of the mean **(A, C)** or Fisher’s exact test **(B, D)**. *P< 0.05; **P< 0.01.

In order to study whether the human commensal bacteria augmented *S. aureus*-induced septic arthritis, mice were inoculated with either *S. aureus* alone or with a mixture of *S. aureus* and *S. epidermidis* or *S. mitis* in a ratio of 1:100. Mice inoculated with a mixture of *S. aureus* together with *S. epidermidis* developed significantly more severe and more frequent clinical arthritis compared to mice inoculated with *S. aureus* alone. Mice infected with *S. epidermidis* did not develop any signs of septic arthritis during the course of infection (**Figures 1A, B**). Similar data were also observed when *S. mitis* was used. Significantly increased severity of clinical arthritis was observed in mice inoculated with combination of *S. aureus* together with *S. mitis* compared to mice inoculated with either *S. aureus* alone or *S. mitis* alone. Also, the frequency of arthritis tended to be higher in mice infected with *S. aureus* and *S. mitis* mixture than mice infected with *S. aureus* alone (**Figures 1C, D**). This strongly suggest that augmentation effect for *S. aureus* septic arthritis can be induced by both *S. epidermidis* and *S. mitis*.

### The Weight Development During Infection Was Not Altered by Addition of Commensal Bacteria

**Figure 2:**
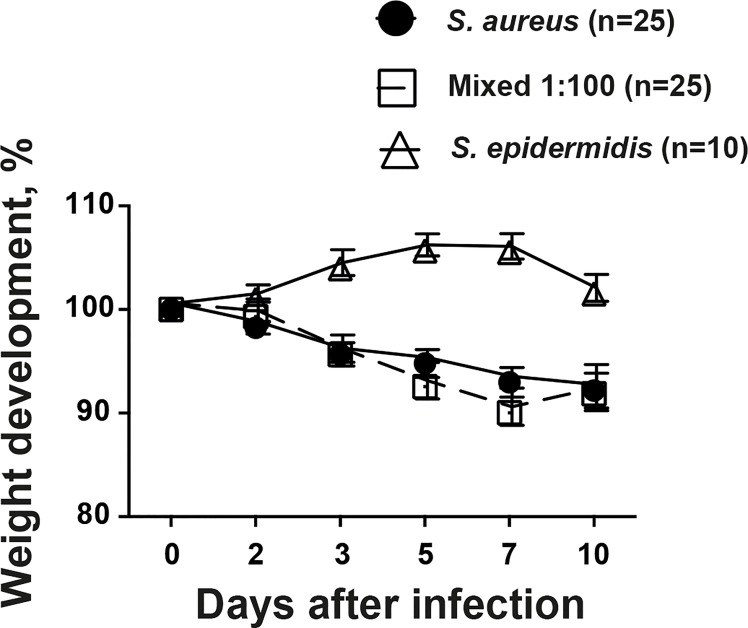
The weight development during infection was not altered by addition of commensal bacteria. NMRI mice intravenously inoculated with either *Staphylococcus aureus (S. aureus)* LS-1 strain (1.2 x 10^6^ CFU/mouse) alone, *Staphylococcus epidermidis (S. epidermidis)* CCUG 61325 (1.2 x 10^6^ CFU/mouse) alone or with *S. aureus* LS-1 strain (1.2 x 10^6^ CFU/mouse) mixed with *S. epidermidis* CCUG 61325 (1.2 x 10^8^ CFU/mouse, denoted Mixed 1:100). The changes in the body weight were followed for 10 days after infection. Statistical evaluations were performed using the Mann–Whitney U test, with data expressed as the mean ± standard error of the mean.

Weight development is a crucial parameter in our animal model describing the general well-being of the animals. Mice inoculated with *S. aureus* alone or mixture of *S. aureus* together with *S. epidermidis* lost significantly more weight compared to mice inoculated with *S. epidermidis* alone throughout the experiment (**Figure 2**). However, no tangible difference was found between mice infected with *S. aureus* alone or mixture of *S. aureus* together with *S. epidermidis.*

### Co-Injection of *S. epidermidis* and *S. aureus* Impairs Ability of Mice to Clear Bacteria in Both Joints and Kidneys

**Figure 3:**
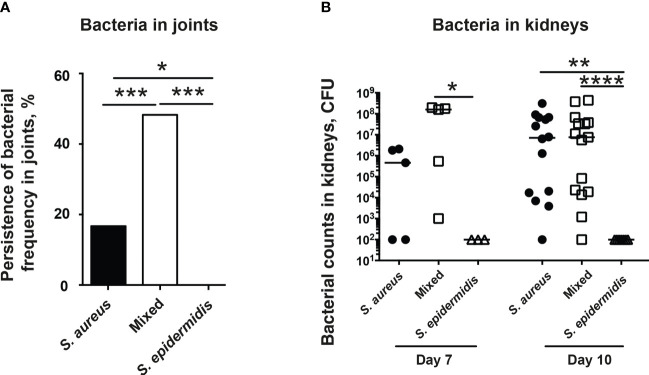
Co-injection of *S. epidermidis* and *S. aureus* impairs ability of mice to clear bacteria in both joints and kidneys. NMRI mice intravenously inoculated with either *Staphylococcus aureus (S. aureus)* LS-1 strain (1.2 x 10^6^ CFU/mouse) alone, *Staphylococcus epidermidis (S. epidermidis)* CCUG 61325 (1.2 x 10^6^ CFU/mouse) alone or with *S. aureus* LS-1 strain (1.2 x 10^6^ CFU/mouse) mixed with *S. epidermidis* CCUG 61325 (1.2 x 10^8^ CFU/mouse, denoted Mixed). **(A)** Persistence of bacterial frequency in joints including wrists, ankles, and knees of the mice (n = 3-5 mice/group) on day 7 post-infection. **(B)** Persistence of bacteria in kidneys of the mice on day 7 post-infection (n = 3-5 mice/group) or on day 10 post-infection (n = 7-15 mice/group). Statistical evaluations were performed using Fisher’s exact test **(A)** or Mann–Whitney U test, with data expressed as the median **(B)**. *P< 0.05; **P< 0.01; ***P< 0.001; ****P< 0.0001.

The accumulation of bacteria in joints is diagnostic for septic arthritis. The increased bacterial counts in kidneys are associated with worse outcome in our *S. aureus*-induced septic arthritis animal model. Thus, we investigated the ability of mice to clear bacteria in both the joints and kidneys. Mice inoculated with the bacterial mixture had significantly higher bacteria recovered in joints compared to mice inoculated with either *S. aureus* or *S. epidermidis* alone (**Figure 3A**). Interestingly, no bacteria were recovered in the joints of mice inoculated with *S. epidermidis* alone whereas bacteria were recovered in 17% of the joints of mice inoculated with *S. aureus* alone (**Figure 3A**).

In contrast, no significant differences in kidney CFU counts were observed in the mice with *S. aureus* alone or a mixture of *S. aureus* and *S. epidermidis* on day 7 and 10 post-infection (**Figure 3B**). No bacteria were found in kidneys from mice infected with high dose of *S. epidermidis* alone (**Figure 3B**).

### Co-Injection of *S. epidermidis* and *S. aureus* Increases Serum Levels of Chemokines in Mice

**Figure 4:**
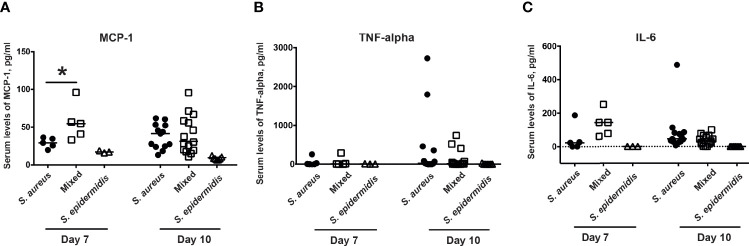
Co-injection of *S. epidermidis* and *S. aureus* increases serum levels of chemokines in mice. The levels of **(A)** monocyte chemoattractant protein 1 (MCP-1), **(B)** tumor necrosis factor alpha (TNF-alpha), and **(C)** interleukin-6 (IL-6) in the blood serum of NMRI mice intravenously inoculated with either *Staphylococcus aureus (S. aureus)* LS-1 strain (1.2 x 10^6^ CFU/mouse) alone, *Staphylococcus epidermidis (S. epidermidis)* CCUG 61325 (1.2 x 10^6^ CFU/mouse) alone or with *S. aureus* LS-1 strain (1.2 x 10^6^ CFU/mouse) mixed with *S. epidermidis* CCUG 61325 (1.2 x 10^8^ CFU/mouse, denoted Mixed) on day 7 post-infection (n = 3-5 mice/group) and on day 10 post-infection (n = 7-15 mice/group). Statistical evaluations were performed using the Mann–Whitney U test, with data expressed as the median. *P< 0.05.

Next step was to investigate whether co-injection of *S. aureus* with *S. epidermidis* increased the serum levels of chemokines and cytokines. Inoculation with the bacterial mixture indeed significantly increased the release of the MCP-1 compared to inoculation with either *S. aureus* or *S. epidermidis* alone on day 7 but not day 10 post-infection (**Figure 4A**). No difference was recorded between the groups with regard to serum levels of the pro-inflammatory cytokines, TNF-α and IL-6 on both day 7 and day 10 post-infection (**Figure 4B, C**).

### *S. epidermidis* Augments *S. aureus*-Induced Septic Arthritis in a Dose-Dependent Manner

**Figure 5:**
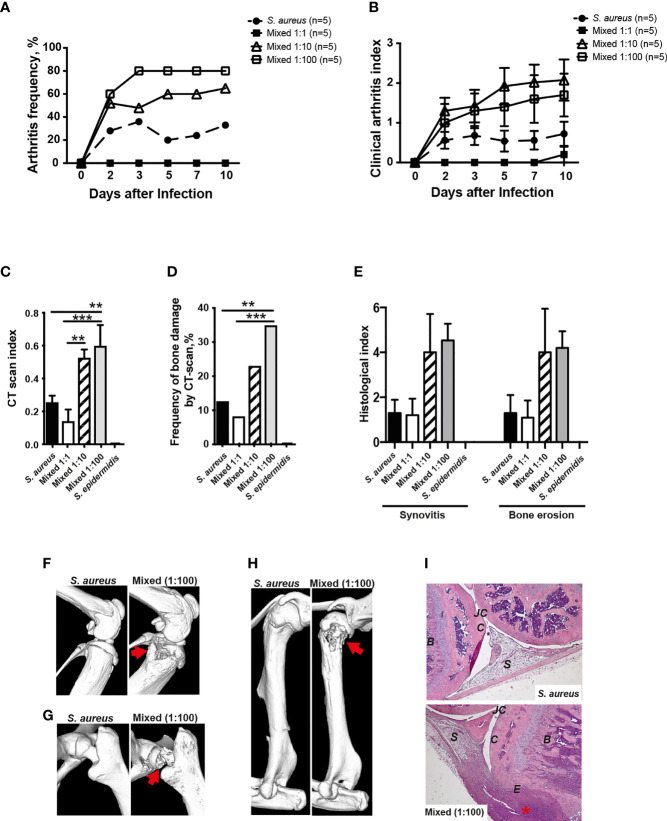
*S. epidermidis* augment S. aureus-induced septic arthritis in a dose dependent manner. NMRI mice were intravenously inoculated with either *Staphylococcus aureus (S. aureus)* LS-1 strain (1.2 x 10^6^ CFU/mouse) alone, *Staphylococcus epidermidis (S. epidermidis)* CCUG 61325 strain (1.2 x 10^6^ CFU/mouse) alone or with *S. aureus* LS-1 strain (1.2 x 10^6^ CFU/mouse) mixed with different concentrations of *S. epidermidis* CCUG 61325 (1.2 x 10^6^ CFU/mouse, denoted Mixed 1:1; 1.2 x 10^7^ CFU/mouse, denoted Mixed 1:10; or 1.2 x 10^8^ CFU/mouse, denoted Mixed 1:100). The frequency of clinical arthritis **(A)**, the clinical severity of arthritis **(B)**, and the radiological accumulative bone destruction scores **(C)** and the frequency of bone destructions **(D)** as evaluated by a microcomputed tomography (μCT)-scan on day 10 post-infection. **(E)** Histopathologic evaluation of synovitis and bone erosions in the joints from all 4 limbs of the 5 groups. Representative μCT-scan images of knee joints **(F)**, hip joints **(G)** and shoulders **(H)** in NMRI mice infected with *S. aureus* illustrating a healthy joint (left panels) and a joint infected with a mixture of *S. aureus* and *S. epidermidis* with severe septic arthritis (right panels) on day 10 post-infection. The erosions are indicated by the arrows. **(I)** Illustrative photomicrographs from NMRI mice infected with *S. aureus* showing a healthy knee joint (upper panel) and NMRI mice infected with a mixture of *S. aureus* and *S. epidermidis* showing a heavily inflamed knee joint with severe bone and cartilage destruction (lower panel) on day 10 post-infection. Histologic staining was performed using hematoxylin and eosin. The asterisk indicates an inflamed synovium. B, bone; C, cartilage; E, erosion of bone and cartilage; JC, joint cavity; S, synovial tissue. Original magnification × 10. Statistical evaluations were performed using the Mann–Whitney U test, with data expressed as the mean ± standard error of the mean **(B, C, E)** or Fisher’s exact test **(A, D)**. **P< 0.01; ***P< 0.001.

In order to study whether the human commensal *S. epidermidis* dose-dependently augments *S. aureus*-induced septic arthritis, mice were inoculated with either *S. aureus* alone or with a mixture of *S. aureus* and *S. epidermidis* in a ratio of 1:1, 1:10 or 1:100. Mice inoculated with a mixture of *S. aureus* and *S. epidermidis* in a ratio of 1:1 did not develop clinical arthritis (**Figures 5A, B**). However, mice inoculated with a mixture of *S. aureus* together with higher doses of *S. epidermidis* (1:10 and 1:100) developed much severe and more frequent clinical arthritis compared to mice inoculated with *S. aureus* alone (**Figures 5A, B**), suggesting that augmentation effect of *S. epidermidis* was dose-dependent. The mice infected with *S. epidermidis* alone did not have any sign of septic arthritis during the whole course of infection.

In line with the data from clinical arthritis, mice inoculated with a mixture of both *S. aureus* and *S. epidermidis* in a ratio of 1:10 and 1:100 significantly developed more severe bone destruction as well as higher frequencies of bone erosions compared to mice inoculated with *S. aureus* alone or a mixture of *S. aureus* and *S. epidermidis* in a ratio of 1:1 on the termination day (**Figures 5C, D**).

A similar trend was seen histologically whereby mice inoculated with *S. aureus* mixed together with *S. epidermidis* displayed more severe synovitis and bone erosion compared to mice inoculated with *S. aureus* alone (**Figure 5E**).

**Figures 5F–H** demonstrate the representative CT images of healthy joints and bone destruction in knees, hips, and shoulders. **Figure I** show the representative histological images of a healthy knee and a knee with severe septic arthritis.

## Discussion

The presence of human commensal organism at the point of infection is known to dramatically increase the *S. aureus* pathogenicity in *S. aureus* sepsis. However, it is still largely unknown whether human commensals augment other types of *S. aureus* infections, such as *S. aureus* septic arthritis. In the current study, for the first time we show that human commensal strains such as *S. epidermidis* and *S. mitis* augment *S. aureus* pathogenicity in inducing septic arthritis in a dose-dependent manner.

Our data demonstrate that human commensals augment *S. aureus* invasion into the joints, causing more joint inflammation and bone destructions. Similarly, significantly more severe abscess formation and higher bacterial load in livers were found in previous reports in the sepsis settings (Boldock et al., 2018). In contrast, we found that bacterial load in the kidneys was not significantly increased by mixed infection. This suggest that augmentation effects by commensal bacteria are somehow tissue-specific, e.g. focal *S. aureus* infections in livers and joints, but not in kidneys were augmented by the commensal strains. It has been shown that augmentation effect of commensal strains on *S. aureus* pathogenicity is mediated through Kupffer cells (Boldock et al., 2018). Kupffer cells, resident liver macrophages, are abundant in liver tissues, serving as the first gatekeeper to eliminate the bacteria, toxins, and microbial debris transported to liver. Monocytes/macrophages are also known to be crucial for induction of septic arthritis. Depletion of monocytes in mice caused reduced severity of septic arthritis in a *S. aureus* septic arthritis mouse model (Verdrengh and Tarkowski, 2000). Lipoproteins, the most pro-inflammatory *S. aureus* component, lost their arthritogenic- as well as the bone resorptive capacities in the monocytes/macrophages depleted mice (Mohammad et al., 2019; Schultz et al., 2022). In addition, the monocytes/macrophages are the precursors of osteoclasts that is one of major bone cells in bone tissues. However, the importance and abundance of macrophages in liver and joints may not explain the organ-specificity of augmentation, as macrophages are also abundant in kidneys. The renal macrophages account for about 50% of total CD45+ leukocytes in mouse kidneys and also found in large numbers in human kidney (Liu et al., 2020). Therefore, the mechanism why augmentation effect by commensals is organ specific is warranted for the further investigations.

In our experimental settings, *S. aureus* were mixed with commensal strains and intravenously injected to mice. The augmentation effect was observed only when ratio of commensal strains and *S. aureus* mixture was higher than 1:1. Does the augmentation niche with right bacteria and right ratio of bacterial mixture exist in humans? *S. aureus* are commonly found on the human skin at different body sites and anterior nares are the reservoir for *S. aureus* skin carriage (Sollid et al., 2014). Up to 30% of the human population are colonized with nasal *S. aureus* (Sakr et al., 2018) and the colonized *S. aureus* in the nares are one of the main sources of *S. aureus* bacteremia (von Eiff et al., 2001). Rapid screening and decolonizing of nasal carriers of *S. aureus* was shown to prevent the surgical-site infections (Bode et al., 2010). The commensal bacteria found in the anterior naris are *S. epidermidis*, *Cutibacterium acnes*, *Dolosigranulum pigrum*, *Finegoldia magna*, *Corynebacterium* spp., *Moraxella* spp., *Peptoniphilus* spp., and *Anaerococcus* spp (Kumpitsch et al., 2019).. *S. epidermidis* that was used in the current study is one of the most predominant opportunistic bacterial species in the naris, suggesting the great possibility of coexistence of both bacteria species in the same location. Importantly, it has been shown that a broad range of microorganisms including *Micrococcus luteus*, *Escherichia Coli*, *Roseomonas mucosa*, and *Saccharomyces cerevisiae* are able to augment the *S. aureus* pathogenicity in sepsis (Boldock et al., 2018; Gibson et al., 2021). Indeed, a study of the overall microbial composition in anterior nare bacterial community in 40 healthy individuals revealed that in most of the cases the other commensal strains were much more prevalent in high abundance than *S. aureus* (Wos-Oxley et al., 2010).

How is the augmentation effect on *S. aureus* septic arthritis mediated by commensal strains? In our current study, both *S. epidermidis* and *S. mitis* were proven to be non-virulent, as neither kidney abscess formation nor weight loss were observed in the mice infected with those commensal strains. It is obvious that commensal strains used in our current study were much more vulnerable to the innate immune killing than *S. aureus*, as no commensal bacteria was recovered from kidneys and joints despite of high infection doses (100 times as *S. aureus* doses). In order to induce a hematogenous septic arthritis, *S. aureus* need to survive the bactericidal components and phagocyte attacks in the blood, disseminate to synovial tissue and finally reach the joint cavity (Jin et al., 2021). For instance, deficiency of neutrophils (Verdrengh and Tarkowski, 1997) or complement 3 (Na et al., 2016) greatly aggravated the *S. aureus* septic arthritis in mice. We hypothesize that as the innate immunity is non-specific, the big number of commensal strains disturbed the phagocytosis and oxygen radical burst by phagocytes against *S. aureus* and therefore significantly more *S. aureus* could survive in blood stream, reach the joint cavity and cause more severe septic arthritis. In addition, the hyperinflammatory responses including enormous leucocyte trafficking and burst of pro-inflammatory cytokines may also contribute to more severe septic arthritis due to increased multitude of infection agents, especially when much higher commensal bacteria were mixed with *S. aureus* during the infections.

Septic arthritis is counted as the most aggressive and devastating joint disease. For patients who have such an infection, even after they have received immediate treatment, the joint damage caused by septic arthritis is often irreversible, leading to permanent joint dysfunction for up to half of the patients (Kaandorp et al., 1995). Furthermore, the emergence of MRSA has severely complicated the available treatment options (DeLeo et al., 2010; Tong et al., 2015). The preventive strategies and novel treatments against septic arthritis are urgently needed. In this study, we have shown that commensal strains such as *S. epidermidis* and *S. mitis* dose-dependently augment *S. aureus* pathogenicity to induce the septic arthritis. This suggests that eradication of both *S. aureus* and other commensal bacteria in high-risk patients for septic arthritis might be a new preventive strategy. It is known that patient screening for *S. aureus* nasal carriers and decolonization of nasal/extranasal sites by topical antibiotics prevent the surgical-site infections (Bode et al., 2010). We need to keep in mind that commensal bacteria, especially microbiota in the guts modulate many vital physiological and metabolic functions as well as the development of immune system (Hooper and Gordon, 2001). The systemic eradication of commensal bacteria may break homeostasis of a balanced microbial ecosystem, which may lead to an unwanted abnormal health situation. Therefore, we propose a similar strategy for high-risk patients to prevent septic arthritis: 1) Systemic decolonization should be avoided and decolonization for nasal sites should be used; 2) Screening for the *S. aureus* nasal carriers should be performed for the patients with higher risk for septic arthritis, such as patients undergoing hemodialysis or peritoneal dialysis (Laupland et al., 2003); 3) Only high-risk patient with *S. aureus* nasal carriage will be treated with the topical antibiotics with broad spectrum covering both *S. aureus* and common commensal.

## Data Availability Statement

The original contributions presented in the study are included in the article/supplementary material. Further inquiries can be directed to the corresponding author.

## Ethics Statement

The animal study was reviewed and approved by The Ethics Committee of Animal Research of Gothenburg. The mouse experiments were performed in accordance with the Swedish Board of Agriculture’s regulations and recommendations on animal experiments.

## Author Contributions

TJ and YF conceived and planned the experiments. YF, MM, and AA carried out the experiments. AA and TJ wrote the manuscript. All authors contributed to the interpretation of the results and provided critical feedback and helped shape the research, analysis and manuscript. All authors contributed to the article and approved the submitted version.

## Funding

This work was supported by the Swedish Medical Research Council [grant number 523-2013-2750 and 2019-01135 to TJ]; grants from the Swedish state under the agreement between the Swedish Government and the county councils, the ALF-agreement [grant number ALFGBG-823941 to TJ]; Rune och Ulla Amlövs Stiftelse för Neurologisk och Reumatologisk Forskning [grant number 2016-075 to TJ]; E och K.G. Lennanders stipendiestiftelse to [AA and MM]; Magnus Bergvalls Stiftelse [grant numbers 2017-01958, 2018-02797 to AA]; Sahlgrenska University Hospital Foundations to [MM] and Institute of Medicine, Gothenburg University. The funders had no role in study design, data collection and analysis, decision to publish, or preparation of the manuscript.

## Conflict of Interest

The authors declare that the research was conducted in the absence of any commercial or financial relationships that could be construed as a potential conflict of interest.

## Publisher’s Note

All claims expressed in this article are solely those of the authors and do not necessarily represent those of their affiliated organizations, or those of the publisher, the editors and the reviewers. Any product that may be evaluated in this article, or claim that may be made by its manufacturer, is not guaranteed or endorsed by the publisher.
